# Overexpression of *Pyrus sinkiangensis* HAT5 enhances drought and salt tolerance, and low-temperature sensitivity in transgenic tomato

**DOI:** 10.3389/fpls.2022.1036254

**Published:** 2022-11-07

**Authors:** Xiaoyan Liu, Aowei Li, Saisai Wang, Chengfang Lan, Yize Wang, Jin Li, Jianbo Zhu

**Affiliations:** Key Laboratory of Agricultural Biotechnology, College of Life Sciences, Shihezi University, Shihezi, China

**Keywords:** HD-ZIP, transcription factor, *Agrobacterium*-mediated method, tolerance, transgenic tomato

## Abstract

The homeodomain-leucine zipper protein HAT belongs to the homeodomain leucine zipper subfamily (HD-Zip) and is important for regulating plant growth and development and stress tolerance. To investigate the role of *HAT5* in tolerance to drought, salt, and low temperature stress, we selected a *HAT* gene from *Pyrus sinkiangensis Yü (Pyrus sinkiangensis* T.T. Yu). The sequences were analyzed using ioinformatics, and the overexpressed tomato lines were obtained using molecular biology techniques. The phenotypes, physiological, and biochemical indexes of the wild-type and transgenic tomato lines were observed under different stress conditions. We found that the gene had the highest homology with *PbrHAT5*. Under drought and NaCl stress, osmotic regulatory substances (especially proline) were significantly accumulated, and antioxidant enzyme activities were enhanced. The malondialdehyde level and relative electrical conductivity of transgenic tomatoes under low temperature (freezing) stress were significantly higher than those of wild-type tomatoes. The reactive oxygen species scavenging system was unbalanced. This study found that *PsHAT5* improved the tolerance of tomatoes to drought and salt stress by regulating proline metabolism and oxidative stress ability, reducing the production of reactive oxygen species, and maintaining normal cell metabolism. In conclusion, the *PsHAT5* transcription factor has great potential in crop resistance breeding, which lays a theoretical foundation for future excavation of effective resistance genes of the HD-Zip family and experimental field studies.

## Introduction

Global warming is a well-known phenomenon; extreme weather changes cause irreversible damage to plant growth. When plants are subjected to abiotic stresses, such as drought, salinity, and low temperature, the germination cycle of seeds will be changed (Huang et al., 2018), reactive oxygen species will be accumulated in large quantities, and the normal metabolic balance will be upset (Girousse et al., 2018; Lodeyro et al., 2021). These will affect normal crop growth and development, and the resulting reduced crop yields and changed crop quality (DaMatta et al., 2018; Rotundo et al., 2019) pose a serious threat to food security and wood supply (Shao et al., 2022). Therefore, over a long evolution, plants have developed complex regulatory mechanisms to defend against habitat changes, with transcription factors being important regulatory factors. Transcription factors play a crucial role in the transcriptional regulation of genes (Wani et al., 2021). They participate in defense responses by interacting with Cis-regulatory elements (CREs) in the promoter regions of various biotic and abiotic stress-responsive genes (Liu et al., 1999), regulating the expression of downstream adversity stress-responsive target genes at the transcriptional level, and prompting plants to regulate at molecular, physiological, and biochemical levels to adapt with various stresses (Toledo-Ortiz et al., 2003; Ma et al., 2022).

Homeodomain leucine zipper (HD-Zip) is a plant-specific transcription factor encoding an HD-Zip protein, which contains a highly conserved HD and a leucine zipper domain (LZ) (Elhiti and Stasolla, 2009). HD motifs can bind DNA specifically, and Zip acts as a dimerization conserved sequence element (Yang et al., 2022). HD-Zip proteins are divided into four subfamilies (I–IV) (Agalou et al., 2008). An increasing number of studies have shown that HD-Zip transcription factors are involved in a wide range of plant life processes, including cell differentiation, growth and development, morphogenesis, and biotic and abiotic stress response (Belamkar et al., 2014; Gong et al., 2019; Sharif et al., 2021).

Vollbrecht et al. (1991) identified the first *HB* genes in plants and isolated them from maize. Subsequently, many *HB* genes were identified and isolated from monocots and dicots. *AtHB12* and *AtHB7* are a pair of paralogous homologs of the HD-Zip I subfamily, both of which can respond to ABA and drought stress (Olsson et al., 2004; Ré et al., 2014). Ectopic expression of *AtHB12* revealed that the transgenic yeast cells showed enhanced tolerance to NaCl, which led to the speculation that *AtHB12* might be involved in NaCl stress response in plants (Shin et al., 2004). The expression level of the *Zmhdz10* gene, a member of the HD-Zip I family, was strongly induced by exogenous ABA and salt stress, and maize lines exhibited stronger drought and salt stress tolerance than wild types (Zhao et al., 2014). The *MtHB1* gene is a member of the HD-Zip I family in alfalfa and is strongly induced by salt stress. The *MtHB1* gene could indirectly inhibit the formation of lateral roots and reduce the degree of damage to the plant by the external environment (Ariel et al., 2010).

The *GmHAT5* gene, a member of the HD-Zip I family in soybean, was greatly elevated by salt stress, and the overexpression of *GmHAT5* significantly enhanced the salt tolerance of *Centella asiatica* (Belamkar et al., 2014; Cao et al., 2016). HD-Zip I transcription factor *MdHB7-like* improves salt tolerance in *Malus domestica* (Zhao et al., 2021). *PsHDZ63* enhances salt stress tolerance in *Populus tremuloides* by combining effective stress elements with transgenic lines to improve reactive oxygen species scavenging capacity (Guo et al., 2021). *AtHB6* may act downstream of the signal transduction pathway in ABI1 and ABI2, mediating drought stress responses (Söderman et al., 1999; Himmelbach et al., 2002). Chickpea (*Cicer arietinum* Linn.) WRKY70 regulates the expression of transcription factor *CaHDZ12*, which confers abiotic stress tolerance to transgenic tobacco to enhance drought and salt tolerance (Sen et al., 2017). *GhHB12*, a cotton stress-responsive HD-Zip transcription factor, negatively regulates cotton resistance by repressing jasmonic acid-response genes (He et al., 2018). The homologous structural domain LZ ranscription factors *HaHB1* and *AtHB13* enhance tolerance to drought and salinity stresses by inducing proteins that stabilize the cell membrane (Cabello et al., 2012; Cabello and Chan, 2012).

Overexpression of the *CaHB12* transcription factor in cotton (*Gossypium hirsutum*) improves drought tolerance (Basso et al., 2021). Sunflower *Hahb-4* is a novel component of the ethylene signaling pathway and induces a significant delay in senescence (Dezar et al., 2005; Manavella et al., 2006). In summary, HD-Zip family genes are widely involved in regulating various growth and developmental processes and abiotic stress responses in plants. HD-Zip genes play an important regulatory role in drought and salt resistance, indicating that this family of genes has great potential to be used in developing new varieties of stress-resistant crops. Strangely, there is almost no information about this HD-Zip family gene in *P. sinkiangensis*, and functional studies are even less available.

Processed tomato (*Solanum Lycopersicum* L.) is an annual or perennial herb in the Solanaceae family and is a warm-loving vegetable. Originally from South America, tomatoes are grown in many parts of the world (Hanssen and Lapidot, 2012); they were rapidly developed in China in the early 1950s, becoming one of the major fruit vegetables. However, global warming has become an unavoidably hot issue and challenge for the international community (Sultan et al., 2019; Sun et al., 2019). Extreme environmental changes have seriously affected crop yield and quality and hindered the potential of agricultural production (Fatima et al., 2020; Epule et al., 2021; Laudien et al., 2022). Therefore, practical guidance is important for improving the resilience of processed tomatoes to adapt with extreme weather changes, which is an urgent issue for agricultural development.

Recent experimental results have shown that HD-Zip genes play important regulatory functions in processes such as drought and salt resistance in crops, which predicts that this family of genes has great potential for application in breeding new varieties of stress-resistant crops (Ariel et al., 2010; Cabello et al., 2017; Zhao et al., 2017). Most HD-Zip-related gene studies have focused on the model plant *Arabidopsis thaliana*. However, *PsHAT5*, a transcription factor of the HD-Zip family, has been little studied. Therefore, in this study, the *PsHAT5* transcription factor encoding the HAT5 protein was cloned from *P. sinkiangensis*. The gene sequence was analyzed using bioinformatics to construct a plant overexpression vector and expression pattern. The transgenic tomato lines were obtained through the *Agrobacterium*-mediated infestation method, and the role of transgenic tomatoes in abiotic stress response (drought, low temperature, and salt) was investigated. This study generated a new series of drought-and salt-tolerant tomato plants. It provided some insights into the molecular regulatory mechanisms of drought and salt tolerance in tomatoes.

## Materials and methods

### Experimental materials and growth conditions

*The P. sinkiangensis used in this study were nursed by the pear variety resource preservation garden of the Institute of Agricultural Science, Second Agricultural Division, Kulle, Xinjiang Uygur Autonomous Region (41°49’34.621’’ N and 86°12’2.317’’E).* “Yaxin 87-5” wild-type tomato seeds Provided by Yaxin Seed Co. Ltd, (Shihezi, China). Wild-type tomato seeds were Sterilized with 10% NaClO for 10 min and then sown on 1/2 MS medium. After 3 days of dark culture and 4 days of light culture, they were grown to obtain sterile seedlings. The hypocotyls of the seedlings were cut, about 1.0 cm long, and cultured in the dark for 2 days in the pre-culture medium. *Agrobacterium tumefaciens* (OD 0.6–0.8) was suspended in MS liquid medium to prepare a recombinant bacterial solution. The pre-cultured tomato stem segments were infested with bacterial fluid for 15 min. Then the bacterial solution on the surface of the explants was sucked dry with filter paper and transferred to a co-culture medium. After 2 days of co-culture, the transformed tomato stem segment callus was transferred sequentially to the screening medium containing 100 mg/mL kanamycin (kan) for about 60 days. After differentiation, the seedlings were transferred to the rooting medium, and the roots were formed after 30 days of induction. Then, they were transplanted into pots after domestication for normal cultivation management. The volume ratio of cultivated soil, peat, vermiculite, and perlite used was 3:1:2. The culture conditions were: 25–28 °C, relative humidity 65–70%, light intensity 8000 lx, light/dark period 16/8 h. MS medium, Sucrose, and Agar strips were purchased from Shenggong Bioengineering Co. Ltd. (Shanghai, China). The medium used in the plant tissue culture process and the regeneration process of the intact plant are both found in the supplementary material (**Supplementary Figures S1-5**).

### Bioinformatics analysis of *PsHAT5* transcription factor

Sequence analysis was done at NCBI (http://www.ncbi.nlm.nih.gov/) and DNAMAN. Multiple comparisons were performed with ClustalW, a phylogenetic tree of *PsHAT5* gene was constructed using MEGA 7.0 with NJ (Neighbor-Joining) method.

### Cloning of *PsHAT5* transcription factor and construction of plant overexpression vector

Total RNA was extracted from the bast *P. sinkiangensis* using RNAisoPlus kit (TaKaRa) containing on-column DNase I according to the manufacturer’s instructions. First-strand cDNA was synthesized from the total RNA using oligo (dT) primers and PrimeScript^®^RTase (TaKaRa). The sequence of white pear (*Pyrus*×*bretschneideri*) PbHAT5 protein (GenBank accession number: XP_009369353.1) published on the NCBI website was used to finally obtain the *PsHAT5* sequence of *P. sinkiangensis* after comparing with the genome database of *P. sinkiangensis* (unpublished) in our laboratory. Based on the sequence information of *PsHAT5*, specific primers were designed using Premier 5.0 software (**Supplementary Table S1**), and PCR amplification of *PsHAT5* transcription factor was performed using cDNA as template. The PCR amplification procedure was: 95°C for 5 min; 95°C for the 30 s, 60°C for 30 s, 72°C for 1 min for 30 s, 35 cycles; And finally, 72°C for 10 min. The purified PCR product was ligated to the pMD^®^19-T Vector and the ligated product was transformed into E. coli DH5-Alpha competent cells. The correctly sequenced T-PsHAT5 recombinant plasmid was inserted into the plant expression vector pCAMBIA2300 by double digestion to construct the pCAMBIA2300-PsHAT5 overexpression vector. After verified by digestion and sequencing, the recombinant plasmid was transferred into *Agrobacterium tumefaciens* GV3101 using the electroshock method for genetic transformation of tomato, Finally, the plant overexpression vector carrying *PsHAT5* transcription factor was constructed.

### Acquisition and identification of transgenic tomatoes

To obtain transgenic tomato plants, wild-type tomato plants were transformed with a plasmid containing PsHAT5 by an *Agrobacterium*-mediated method. The specific methods are described in the “2.1 Experimental materials and growth conditions” section. First, we extracted DNA from the leaves of wild-type tomatoes and transformed tomato plants using the CTAB method, and used the leaf DNA of wild-type tomato plants as a negative control and the recombinant plasmid as a positive control for their PCR identification. Secondly, we used EasyPure Plant RNA Extraction Kit (Transgen Biotech, China) to extract total tomato RNA by referring to the operating instructions of the kit. First-strand cDNA was generated from RNA by using a reverse transcription system (Takara Biotechnology, Kusamatsu, Japan). The expression levels of *PsHAT5* transcription factors were determined by qPCR in 10 μL reactions using the Roche LightCycle^®^ 480 System (Salt Lake City, UT, USA) and SYBR Green Real-Time PCR Master Mix (KAPA Biosystems, Wilmington, DE, USA). Contain 5 μL 2× SYBR Green Real MasterMix (SYBR Green, Applied Biosystems), 0.5 μL cDNA, 0.5 μL each of upstream and downstream primers for *PsHAT5* transcription factor (**Table S1**), and the rest was made up to 10 μL with ddH_2_O. The amplification procedures were as follows: 95°C for 5 min, 95°C for the 30 s, 60°C for 30 s, 72°C for 30 s, 25 cycles; Finally, 72°C for 10 min, Using GAPDH as an internal reference. We selected T0 generation tomatoes for PCR identification and T1 tomatoes for qPCR identification (146bp), and through analysis, we selected two independent T2 generation homozygous strains (OE-2, and OE-4) for subsequent experiments.

### Evaluation of stress tolerance

We selected 4-week-old wild-type and transgenic tomatoes of uniform size and good growth status from the same period and subjected them to drought and low temperature as well as salt stress treatments. Drought stress was performed as follows: Wild-type and transgenic tomatoes were watered fully and no longer watered during the period of continuous drought. Samples were taken on the 14th day and normal-watered tomatoes were used as control. Then, the tomatoes were watered for 7 d after drought stress. The specific methods of salt stress were as follows: wild-type and transgenic tomato plants of the same growth period were taken and watered with 200 mL of 200 mM NaCl and 250 mM NaCl solution every 3 d for 14 d. The control group was replaced with an equal amount of water; after the salt stress was over, the recovery test was conducted with normal watering for 7 d. The low temperature stress treatments were as follows: wild-type and transgenic tomatoes were subjected to low temperature stress treatments at 4°C for 8 h and -2°C for 6 h, respectively, in an artificial low temperature climatic chamber, using the 25°C treatment as a control. After the stress, 25°C was recovered. The growth status of wild-type and transgenic tomatoes at each treatment stage was observed and recorded by taking photographs with a camera. For each of the above stress treatments, three plants per treatment and three replications were performed.

### Determination of relevant physiological and biochemical indexes

After each stress treatment, we tried to select tomato leaves from the same parts for subsequent physiological and biochemical indexes. Relative water content (RWC) was determined according to the method of Jun Cui et al. (Cui et al., 2018). Relative electrolyte leakage (REL) was determined according to the method of Wang et al. (Wang et al., 2014). MDA content was assessed according to the method of Jun Ma et al. (Ma et al., 2015). We used the method of Liu et al. (Liu et al., 2020) to determine the soluble sugar, soluble protein, and proline contents. For determining antioxidant enzyme activity: 0.5 g of fresh tomato leaves were accurately weighed on an analytical balance and ground in 50 mM/L cold PBS buffer (pH 7.8) with 1.0 mM/L EDTA-Na_2_ and 2% (w/v) polyvinyl pyrrolidone (PVP) in an ice bath to make a homogenate, followed by centrifugation at 4000 r at 4°C. After centrifuging the samples for 15 min, the supernatant was retained as the crude extract for the enzyme activity assay. Specific activity assays of superoxide dismutase (SOD), peroxidase (POD), and catalase (CAT) were carried out according to the description of Muhammad Ali Khan et al. (Khan et al., 2022). Three biological replicates were performed for each indicator.

### Statistical analysis

All measured data were subjected to three biological replicates, and statistical analysis was completed using SPSS and GraphPad Prism 8.3 software. * P < 0.05 (weak significant difference), ** P < 0.01 (significant difference), *** P < 0.001 (strong significant difference), and **** P < 0.0001 (very strong significant difference) are used to indicate significance. In addition, NS indicates that there is no significant difference between the two.

## Results

### Bioinformatics analysis of *PsHAT5* transcription factor

**Figure 1:**
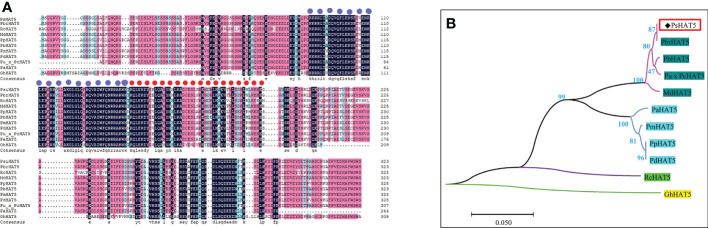
Homologous sequence alignment and phylogenetic analysis of *PsHAT5* transcription factor. **(A)** Sequence comparison results of *PsHAT5* with homologous proteins in other species (The purple circles represent the HD domain, and the red circles represent the LZ domain.). **(B)** Phylogenetic tree of *PsHAT5* (The phylogenetic tree was built using MEGA 7.0 by the Neighbor-Joining method with 1000 bootstrap replicates).

*PsHAT5* had the highest homology with white pear *PbrHAT5* (GenBank: XM_009371078.3) using BlastP analysis on the NCBI website, indicating that the *PsHAT5* transcription factor has the closest genetic relationship with white pear *PbrHAT5*. The next highest homology was with *Pyrus betulifolia bunge* (Chr6.g51268), *Pyrus ussuriensis x Pyrus communis* (KAB2633474.1), *Malus domestica* (NM_001328891.1), *Prunus avium* (XP_ 021808346.1), *Prunus mume* (XM_008241624.1), *Prunus persica* (XP_007209345.1), *Prunus dulcis* (XM_034359186.1), *Rosa chinensis* (XM_024315270.2), and *Gossypium hirsutum* (XP_040939127.1). These species have high homology of HAT5 protein, and all have two highly conserved functional structural domains (**Figure 1A**). The above results strongly suggest that the *PsHAT5* transcription factor is a member of the HD-Zip transcription factor family. The final cloned cDNA sequence of 1089 bp in length was analyzed using DNAMAN software, and the fragment was found to contain a 975 bp open reading frame (ORF) encoding 324 amino acids. A phylogenetic tree of different HAT5 proteins was constructed using MEGA 7.0 software (**Figure 1B**).

### Construction of plant overexpression vectors and acquisition of transgenic tomatoes

**Figure 2:**
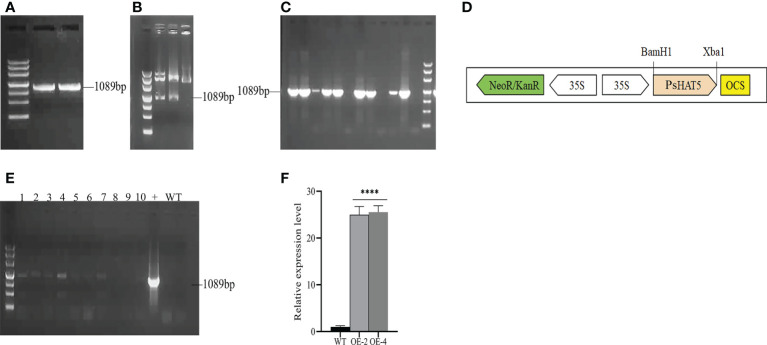
**(A-D)**: The plant Overexpression vector construct of *PsHAT5*. **(A)** Cloning of *PsHAT5* gene. **(B)** The recombinant plasmid pCAMBIA2300-35S-PsHAT5-Nos was identified by restriction enzyme digestion. **(C)** Identification of *PsHAT5* gene by *Agrobacterium tumefaciens* GV3101. **(D)** pCAMBIA2300-35S-PsHAT5-Nos Expression Frame. **(E, F)** PCR identification of transgenic tomatoes with PsHAT5 gene. **(E)** PCR identification of transgenic plants. Numbers 1-10 indicate individual transgenic plant lines, the “+” plasmid was used as a positive control, and WT were used as negative controls. **(F)** Relative expression levels of transgenic tomatoes. (****P < 0.0001 for comparisons between the transgenic lines and wildtypeplants by Student’s t-tests).

To generate the pCAMBIA2300-PsHAT5 recombinant plasmid, the *PsHAT5* ORF was cloned into the pCAMBIA2300 vector under the control of the CaMV35S promoter. The insert was released from pMD19-T- PsHAT5 through BamH I and Xbal I digestion and then ligated into pCAMBIA2300-35S-PsHAT5-Nos. The construct was introduced into *Agrobacterium* GV3101 by electroporation to complete the construction of the overexpression vector (**Figures 2A–D**). *PsHAT5* transgenic tomato plant lines were generated and selected to assess the significance and effect of the *PsHAT5* transcription factor on the physiology of transgenic tomato plants under low temperature, drought, and salt stress. *Agrobacterium*-mediated infection of wild-type tomatoes and screening of T0 transgenic seeds on MS medium containing 200 mg/L kanamycin was used to obtain *PsHAT5*-transformed tomato plants. The genomic DNA of tomato leaves was extracted using the CTAB method, and PCR was performed on the transformed tomato plants using DNA as a template. The agarose gel electrophoresis results showed that eight transgenic tomato plants had been obtained (**Figure 2E**) after a preliminary determination of the specific band at 1089 bp. Finally, we selected OE-2 and OE-4 overexpressing transgenic tomato plants for qPCR analysis, which showed that the relative expression levels of *PsHAT5* in OE-2 and OE-4 were significantly higher than those in wild-type tomatoes (**Figure 2F**). Based on these results, we determined that the *PsHAT5* transcription factor had been successfully transformed into tomato plants, and we selected OE-2 and OE-4 strains for subsequent experiments.

### Overexpression of *PsHAT5* transcription factor enhances drought tolerance in transgenic tomato

**Figure 3:**
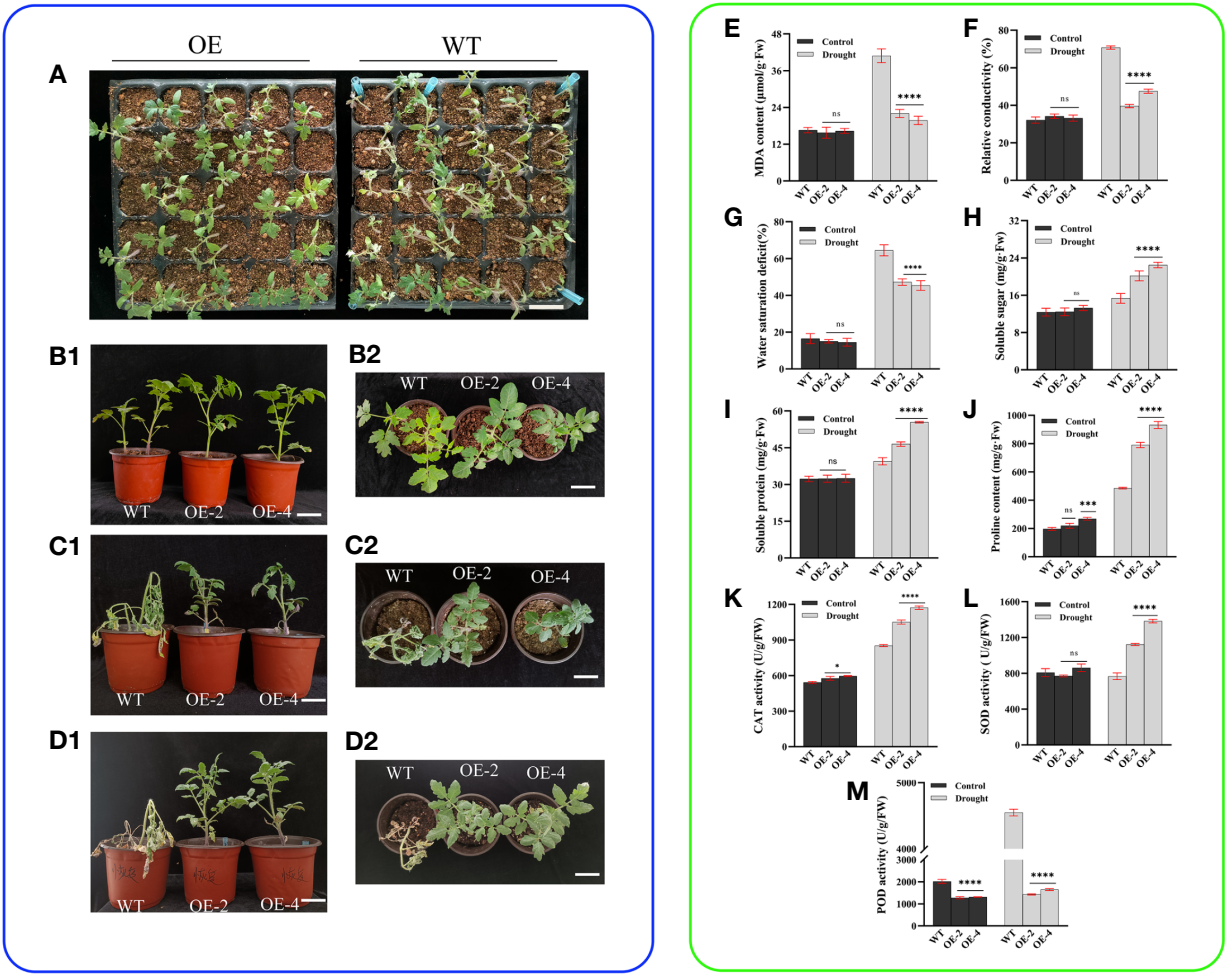
Phenotypic and physiological data of wild-type and transgenic tomatoes under drought stress. **(A)** The overall growth of wild type and transgenic tomatoes seedling trays under drought stress for 7 days. **(B1)** (Main view) and **(B2)** (top view) Growth status of wild-type and transgenic tomatoes at 4 weeks of age after normal watering. **(C1)** (Main view) and **(C2)** (top view): Morphological differences of 4 weeks tomatoes under drought stress for 16 days. **(D1)** (Main view) and **(D2)** (top view): The growth status of C1or C2 after 7 days of rehydration. **(E)** MDA content. **(F)** Relative conductivity content. **(G)** Water saturation deficit. **(H)** Soluble sugar content. **(I)** soluble protein content. **(J)** Proline content. **(K)** CAT activity. **(L)** SOD activity. **(M)** POD activity. Data are means ± SD of three replicates. (7.2 cm inner diameter, 11.5 cm high). (Ns > 0.05, *P < 0.05, ***P < 0.001, and ****P < 0.0001 for comparisons between the transgenic lines and wild-type plants by Student’s t-tests).

In our indoor cultivation experiments, we found that transgenic tomatoes sown in seedling trays showed better drought tolerance than the wild-type (**Figure 3A**). To further investigate the role of the *PsHAT5* transcription factor in drought, we subjected the obtained transgenic and wild-type tomatoes to drought stress treatment (**Figures 3B1, B2**). It was found that the growth of wild-type and transgenic tomatoes was almost identical when watered normally, and there were almost no significant differences between wild-type and transgenic tomato plants when analyzed for physiological and biochemical indicators. When the drought treatment was applied at 16 days, wild-type tomato leaves showed non-extreme wilting and water loss in phenotype, with severe wilting of the apical part and subsequent loss of some lateral branches. Still, the transgenic tomatoes remained upright with bright green leaves (**Figures 3C1, C2**). The results of the physiological and biochemical data showed that the malondialdehyde content, water saturation deficit, and relative conductivity of wild-type tomatoes were significantly higher, and the content of osmoregulatory substances (soluble sugars, soluble proteins, and proline) was significantly lower than those of transgenic tomatoes (**Figures 3E–J**). Malondialdehyde, the final breakdown product of membrane lipid peroxidation, and relative conductivity reflecting the permeability of cell membranes are important indicators of damage to the membrane system, and the levels of malondialdehyde and conductivity can directly reflect the extent of plant damage from adversity. The short-term changes in water saturation deficit reflect the regulation mechanism between various water saturations and affect the photosynthesis of plants. The larger the value, the stronger the resistance of plants to dehydration. The increase in the content of osmoregulatory substances such as soluble sugars, soluble proteins, and proline maintains the dynamic balance of the internal and external cellular environment.

CAT, SOD, and POD are the main enzymes in the antioxidant system, and they act synergistically to maintain the free radical content in plants at homeostatic levels. Notably, the enzyme activities of CAT and SOD were significantly higher in transgenic tomatoes than in the wild-type, indicating that the transgenic strains were less damaged by membrane lipid peroxidation (**Figures 3K–M**). The level of POD activity can reflect plant growth and development and intrinsic metabolism and serve as a physiological indicator of tissue aging. We found that POD enzyme activity was significantly lower than in the wild-type under normal watering treatment and drought treatment, probably due to POD’s ability to convert certain carbohydrates contained in tissues into lignin, increasing lignification and delaying aging. Thus, the antioxidant enzyme system played an important role throughout the drought. Subsequently, we conducted re-watering tests on wild-type and transgenic tomatoes after drought stress. After 7 days of re-watering, wild-type plants did not recover and gradually wilted and died, while transgenic tomato strains had bright green leaves, upright stalks, and vigorous growth, with an increase in plant height and stem thickness of 2.2 cm and 41 mm (average), respectively (**Figures 3D1, D2**). These findings indicate that the overexpression of the *PsHAT5* transcription factor significantly improved drought tolerance in transgenic tomatoes.

### Overexpression of *PsHAT5* transcription factor enhances salt tolerance in transgenic tomato

**Figure 4:**
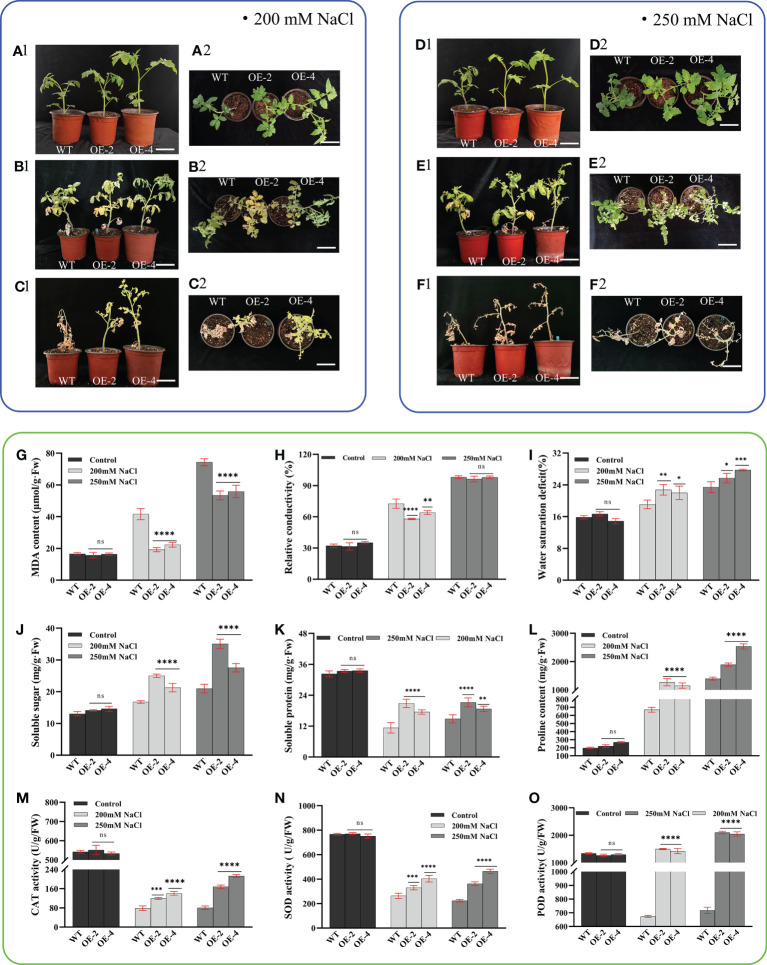
Phenotypic and physiological data of wild-type and transgenic tomatoes under NaCl stress (200mM NaCl and 250mM NaCl). **(A1)** (Main view) and **(A2)** (Top view): The growth status of wild-type and transgenic tomato at 4 weeks of age under normal watering. **(B1)** (Main view) and **(B2)** (Top view): Wild-type and transgenic tomato growth status after 14 days of 200 mM NaCl treatment. **(C1)** (Main view) and **(C2)** (Top view): The growth status of B1 or B2 after 7 days of normal watering. **(D1)** (Main view) and **(D2)** (Top view): The growth status of wild-type and transgenic tomato at 4 weeks of age under normal watering. **(E1)** (Main view) and **(E2)** (Top view): Wild-type and transgenic tomato growth status after 14 days of 200 mM NaCl treatment. **(F1)** (Main view) and **(F2)** (Top view): The growth status of E1 or E2 after 7 days of normal watering. **(G)** MDA content. **(H)** Relative conductivity content. **(I)** Water saturation deficit. **(J)** Soluble sugar content. **(K)** soluble protein content. **(L)** Proline content. **(M)** CAT activity. **(N)** SOD activity. **(O)** POD activity. Data are means ± SD of three replicates. (7.2 cm inner diameter, 11.5 cm high). (Ns > 0.05, *P < 0.05, **P < 0.01, ***P < 0.001, and ****P < 0.0001 for comparisons between the transgenic lines and wild-type plants by Student’s t-tests).

To investigate whether the *PsHAT5* transcription factor can regulate the tolerance of tomato plants to salt stress, we conducted a salt tolerance assay. The results showed that phenotypically, both wild-type and transgenic tomatoes grew vigorously and branched well under normal watering conditions (**Figures 4A1, A2; D1, D2**). When treated with 200 mM and 250 mM NaCl stress, the leaves of wild-type and transgenic plants yellowed, but wild-type tomato leaves showed a large number of yellow dead spots, and after rehydration, wild-type and transgenic tomatoes showed very different growth states (**Figures 4B1, B2; E1, E2**). After rehydration of tomatoes after 250 mM NaCl stress, the vast majority of leaves and petioles of both wild-type and transgenic tomatoes fell off, but the main stems of transgenic tomatoes still survived (**Figures 4C1, C2; F1, F2**). After 7 days of rehydration, the transgenic tomato could maintain a good growth state after 200 mM NaCl stress, while the control plants were wilted and greened until death.

In terms of physiological indicators, malondialdehyde content can directly reflect the level of cell membrane damage in plants under stress conditions. We found that the malondialdehyde content of wild-type tomatoes was higher than that of transgenic tomatoes under 200 mM NaCl treatment, indicating that wild-type and transgenic tomato plants were damaged to different degrees after NaCl stress treatment. With the increase in salt treatment concentration, cell membrane damage remained significantly higher in wild-type tomatoes than in transgenic tomatoes under 250 mM treatment. Analysis of the relative conductivity data revealed that the relative conductivity content of wild-type tomatoes was significantly higher than that of transgenic tomatoes under 200 mM and 250 mM NaCl stress. However, at 250 mM treatment, the relative conductivity content of both wild-type and transgenic tomatoes exceeded 90% (**Figures 4G, H**). This indicated that the cell membrane was severely damaged, membrane permeability was almost lost, and intracellular electrolytes leaked out, thus contributing significantly to the eventual death of wild-type and transgenic tomatoes in subsequent recovery experiments.

The water saturation deficit did not differ between wild-type and transgenic tomatoes under NaCl stress but was increased compared to tomatoes treated with normal watering; soluble sugars and proline were important physiological osmoregulatory substances (**Figures 4I–L**). Under NaCl stress, the content of soluble sugars increased significantly in the transgenic tomatoes compared to the wild-type, which improved the water retention capacity of plant cells; the accumulation of proline was also significantly higher in the transgenic plants compared to the wild-type, which effectively improved the ability of cells to absorb and retain water in salt stress; soluble protein is not only a nutrient but also an important osmoregulator. The increase and accumulation of soluble protein in plants can also improve the water retention capacity of cells. Under normal watering treatment, wild-type and transgenic tomato plants maintained a high soluble protein content, which decreased with increasing salt stress concentration in both wild-type and transgenic plants but remained significantly higher in transgenic plants than in wild-type. This indicated that the osmoregulatory substances, soluble sugars, proline, and soluble protein, play an important role in conferring salt tolerance in tomatoes.

POD, CAT, and SOD are all important members of the plant antioxidant enzyme system. POD enzyme activity increased with increasing NaCl stress concentration but overall was significantly higher in transgenic tomatoes than in the wild-type. CAT and SOD enzyme activities decreased in wild-type and transgenic tomatoes under NaCl stress compared to normally watered tomatoes. However, CAT and SOD enzyme activities were significantly higher in transgenic tomatoes than in the wild-type (**Figures 4M–O**). Under NaCl stress, the accumulation of oxygen radicals was excessive, the degree of membrane lipid peroxidation was severe, more lipid membrane peroxidation products were also produced, and the structure and integrity of membranes were disrupted, causing physiological and biochemical disorders and leading to plant death. This may also be one of the reasons for the poor growth status and eventual death of tomatoes after rehydration. The above findings suggest that the overexpression of the *PsHAT5* transcription factor enhances salt tolerance in transgenic tomatoes.

### Overexpression of the *PsHAT5* transcription factor increases the low temperature sensitivity of transgenic tomatoes (mainly freeze sensitive)

**Figure 5:**
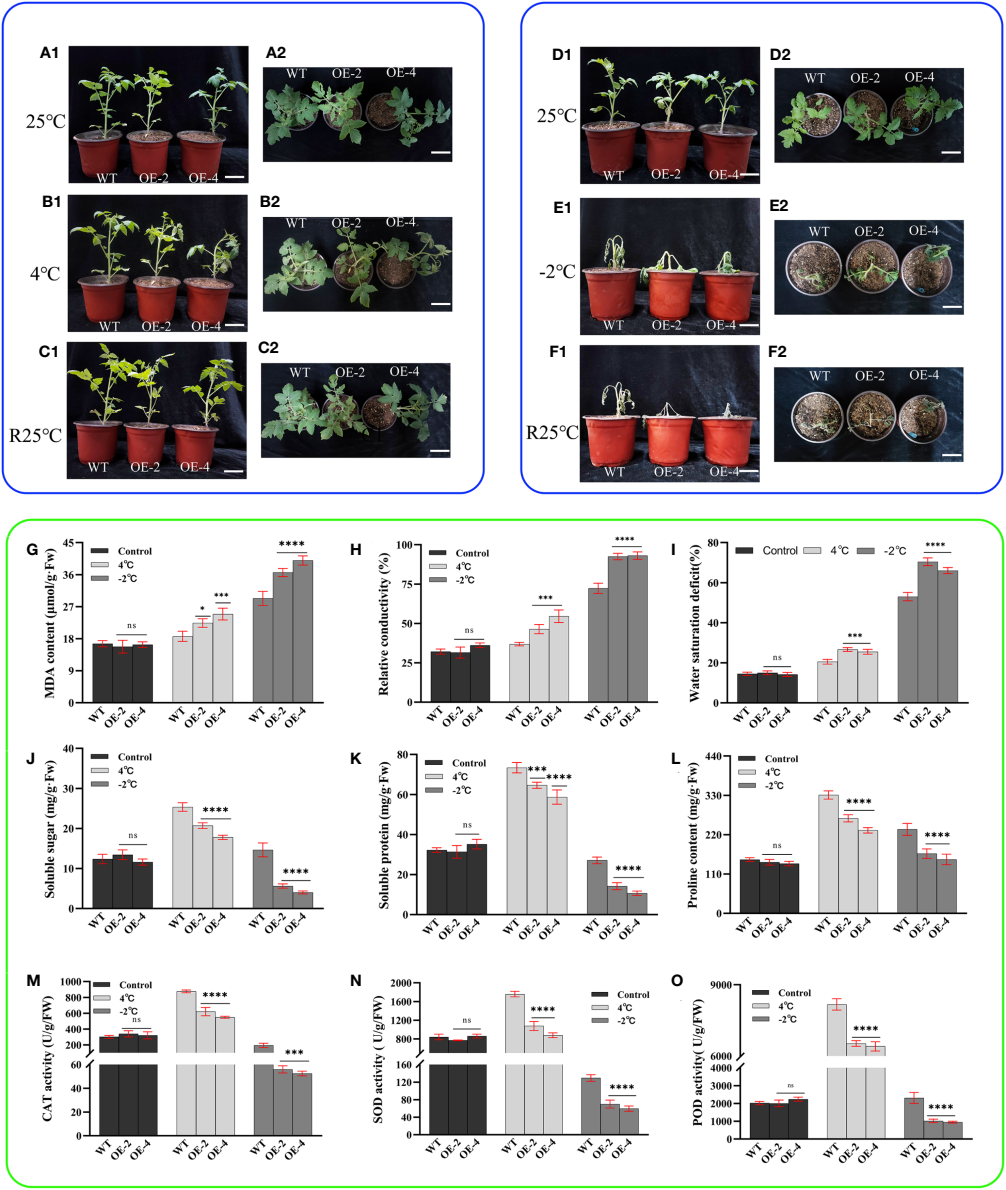
Phenotype and physiology of wild-type and transgenic tomatoes under low temperature (cold and freezing) stress. **(A1)** (Main view) and **(A2)** (Top view): Growth status of wild-type and transgenic tomatoes 4-week-old at room temperature. **(B1)** (Main view) and **(B2)** (Top view): The growth status of wild-type and transgenic tomatoes treated at 4°C for 8h. **(C1)** (Main view) and **(C2)** (Top view): The growth status of B1or B2 after 24 h of room temperature recovery. **(D1)** (Main view) and **(D2)** (Top view): Growth status of wild-type and transgenic tomatoes 4-week-old at room temperature. **(E1)** (Main view) and **(E2)** (Top view): The growth status of wild-type and transgenic tomatoes treated at -2°C for 6h. **(F1)** (Main view) and **(F2)** (Top view): The growth status of E1or E2 after 24 h of room temperature recovery. **(G)** MDA content. **(H)** Relative conductivity content. **(I)** Water saturation deficit. **(J)** Soluble sugar content. **(K)** soluble protein content. **(L)** Proline content. **(M)** CAT activity. **(N)** SOD activity. **(O)** POD activity. Data are means ± SD of three replicates. (7.2cm inner diameter, 11.5cm high.). (Ns > 0.05, *P < 0.05, ***P < 0.001, and ****P < 0.0001 for comparisons between the transgenic lines and wild-type plants by Student’s t-tests).

As a cold-sensitive crop, a short period of low temperature stress can induce significant phenotypic damage changes in tomatoes. We subjected the transgenic tomatoes to low temperature stress to investigate whether the PsHAT5 transcription factor plays a regulatory role at low temperatures. At room temperature, wild-type and transgenic tomatoes grew almost identically with bright green leaves (**Figures 5A1, A2, D1, D2**). After 8 h of cold treatment at 4°C, phenotypically, transgenic tomato leaves were dehydrated with curled edges and drooping tips. In contrast, wild-type tomatoes were almost identical to their pre-treatment state without wilting. Thus, wild-type tomatoes showed stronger cold tolerance (**Figures 5B1, B2**). After 24 h of recovery at room temperature, both wild-type and transgenic tomatoes returned to their pre-treatment growth status (**Figures 5C1, C2**).

When the -2°C freezing treatment was applied for 6 h, the transgenic tomato leaves lost water severely and had a touch of freezing. The top leaves of the plants were completely frozen and showed many obvious dead spots, while the wild-type tomato leaves also showed partial water loss with wilting and yellow dead spots (**Figures 5E1, E2**). After a 24-h room temperature recovery experiment, the transgenic tomato plants were severely dehydrated and died eventually. However, the main stems of the wild-type tomato plants remained upright with some green leaves (**Figures 5F1, F2**). This indicates that the freezing sensitivity of transgenic tomato is increased. From the physiological and biochemical indices, malondialdehyde content, relative conductivity, and water saturation deficit all increased to decreasing treatment temperature. However, wild-type tomatoes were significantly lower than transgenic tomatoes, and when the temperature dropped to -2°C, the relative conductivity of transgenic tomatoes reached more than 90% (**Figures 5G–I**).

Soluble sugar, soluble protein, and proline contents all showed increasing trends with decreasing treatment temperatures. The contents were significantly higher in wild-type tomatoes than in transgenic tomatoes (**Figures 5J–L**). When the temperature decreased to -2°C, it showed a decreasing trend, but wild-type tomatoes were significantly higher than transgenic tomatoes. Soluble sugars, soluble proteins, and proline all play an important role in the osmoregulation of plants as osmoregulatory substances, thus alleviating the damage to plant biofilms caused by low temperature environments; they show a certain trend of complementarity with each other. There was no significant difference in the enzyme activities of CAT, SOD, and POD among the plants at room temperature (**Figures 5M–O**). As the treatment temperature decreased, the CAT, SOD, and POD activities of wild-type and transgenic tomato plants first increased and then decreased. However, the overall trend was that the enzyme activity of wild-type tomatoes was significantly higher than that of transgenic tomatoes. The above results indicated that introducing the *PsHAT5* transcription factor increased the sensitivity of tomatoes under low temperature treatment.

## Discussion

HD-Zip transcription factors are a class of transcription factors encoding homologous heterotypic domain LZ proteins unique to plants that recognize and bind to specific regulatory elements in the promoter regions of target genes to regulate plant growth and development processes involved in plant responses to adverse environmental conditions. Adverse conditions can affect plant growth and development and directly determine plant survival in severe cases. In this study, we increased the drought and salt tolerance of transgenic tomatoes by overexpressing the *PsHAT5* transcription factor into tomatoes to enhance the activity of antioxidant enzymes (CAT, SOD, POD) system and increase the water retention capacity of osmoregulatory substances (soluble sugars, soluble proteins, proline content). Conversely, CAT, SOD, and POD enzyme activities were significantly reduced in low temperature stress, especially freezing stress. The decrease in enzyme activities was greater in transgenic tomatoes, increasing the freezing sensitivity of overexpressed tomatoes.

Relative conductivity is an important physiological and biochemical index reflecting the condition of the plant membrane system. When plants are subjected to adversity or other damage, the cell membrane tends to rupture, and the intracellular material exudates, increasing relative conductivity (Du et al., 2019). Therefore, relative conductivity can reflect the osmoregulatory ability of the plasma membrane under abiotic stress (Hasanuzzaman et al., 2020). Therefore, conductivity studies have become an accurate and practical method to identify the strength of plant stress resistance in crop resistance cultivation and breeding (Ma et al., 2017). In our study, after drought, salt, and low temperature stress treatments, the relative conductivity content of wild-type and transgenic tomatoes increased significantly after each stress treatment compared with those treated normally. Also, 250 mM NaCl and -2°C treatments directly exceeded 90% of the relative conductivity content. The plants did not recover even after 7 days of rehydration at a later stage but gradually dried out and died. The higher the relative conductivity, the greater the permeability of the cell membrane (Li et al., 2014), and the greater the damage to the plant, which exceeds the recovery capacity of the plant. Even if irrigation is carried out, recovery is difficult, and the plant will eventually dry up and die. The research of Menachem Moshelion, Tom Eeckhaut, and Leon V Kochian et al. (Eeckhaut et al., 2013; Kochian et al., 2015; Moshelion et al., 2015) also confirms this point. Their studies pointed out that, in general, abiotic stress in the short term would make the plant leaves lose their green color and wilt, and with timely rehydration, the plant would quickly return to its original growth condition. Different plants have significantly different stress tolerance and behave differently to external drought (Dietz, 2014; Seo, 2014).

In this experiment, the MDA accumulation of transgenic tomatoes after drought and NaCl treatment was relatively less, and the relative conductivity was significantly lower than that of the wild-type, which significantly alleviated the degree of oxidative damage and increased the responsiveness of transgenic tomatoes to drought and salt. MDA is a final product of unsaturated fatty acid peroxidation in phospholipids (Bokhorst et al., 2010; Bresson et al., 2018). Its content can reflect lipid peroxidation or cell membrane damage in plant tissues, affecting protein synthesis (Khan et al., 2021b). The results of Zhao H, Liu Z, and Zhang T et al. also demonstrated that malondialdehyde content accumulates substantially after abiotic stress in plants, aggravating the degree of membrane lipid peroxidation (Zhang et al., 2020; Liu et al., 2022; Zhao et al., 2022). When plants are subjected to osmotic stress, many plants accumulate proline in response to these stresses (Chen et al., 2013).

Proline, a water-soluble amino acid (Naliwajski and Skłodowska, 2021), is a typical osmoprotectant that prevents dehydration and denaturation of proteins under osmotic stress conditions, maintains the osmotic balance inside and outside the cell, and prevents water dissipation. For plants, proline accumulates over time and effectively has the ability of osmotic protection (Aganchich et al., 2009; Ben Ahmed et al., 2009). Our study also found that plants can reduce the osmotic potential of cells under stress by accumulating proline, soluble sugar, and soluble protein, enhancing the water retention and water absorption capacity of plant cells and alleviating the damage of abiotic stress to plants. The accumulation of these substances in transgenic tomatoes is significantly higher than that of wild-type tomatoes, giving them more drought and salt tolerance. Munsif et al. (2021) also revealed that abiotic stress significantly reduced the accumulation of substances such as soluble sugars and proteins.

When plants suffer from stress, the plant antioxidant enzyme system can activate self-defense function, balance reactive oxygen species metabolism, protect the membrane system, and enhance the resilience of plants (Miller et al., 2010; Raza et al., 2021; Sun et al., 2021). Our results showed that the changes in CAT, SOD, and POD enzyme activities did not show obvious synchronization under drought stress. The activities of CAT and SOD were significantly higher than those of the wild type, while the activity of POD was significantly lower than that of the wild type. The possible reason is that these three enzymes may have a coordinated relationship in plant adaptation to stress, which was also proved by Liu et al. (Khare et al., 2015; Liu et al., 2016). Under most environmental stresses, the change in antioxidant enzyme activity appears “first rising then falling” with the increase of stress intensity (Farhangi-Abriz and Torabian, 2017; Khan et al., 2021a; Wang et al., 2021). This is different from our results.

Our results showed that the levels of antioxidant enzymes in wild-type and transgenic lines showed a downward trend under NaCl and freezing stress. In this regard, we have made a possible conjecture and speculation. As a whole, the physiological functions of plants are interrelated, and the antioxidant enzyme system is only one aspect of plant adaptation and resistance to adversity. After exceeding the endurance capacity of cells, prolonged and excessive environmental stress will lead to a deeper degree of membrane lipid peroxidation and enhanced damage to cells, so the activity of antioxidant enzymes will decrease (Sihag et al., 2019; Li et al., 2020). The experiment of Fatma Bejaoui et al. also showed that the change in antioxidant enzyme activity was related to membrane damage index, plant growth cycle, photosynthesis, and secondary metabolite synthesis (Bejaoui et al., 2016; Tian et al., 2018; Liu et al., 2019). It also confirmed the reliability of our research results. These results indicated that the PsHAT5 transcription factor increased drought tolerance, salt tolerance, and low temperature sensitivity in transgenic tomatoes.

## Conclusion

**Figure 6:**
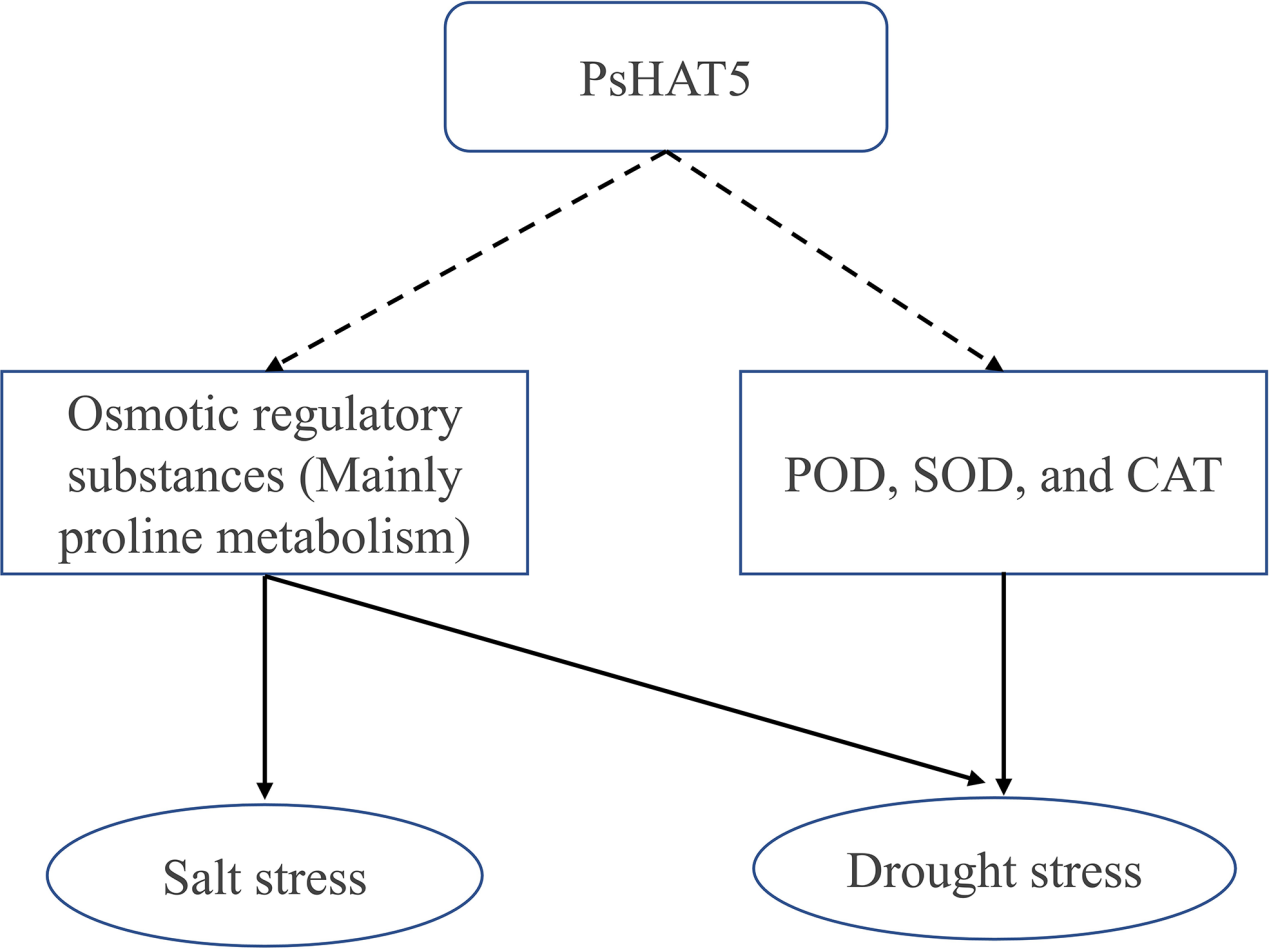
A pictorial representation of the role of *PsHAT5* transcription factors in the regulation of drought and salt stress in tomatoes. Dotted lines indicate events that have not yet clearly occurred and are subject to further study, and the solid lines represent events that occur.

In this study, we cloned the HD-Zip family transcription factor *PsHAT5* from *P. sinkiangensis* and functionally validated it in overexpressing tomatoes. Phenotypic observations and related stress resistance physiological and biochemical indices revealed that overexpression of transgenic tomatoes increased resistance to drought and salt stress by increasing osmoregulatory substances and enhancing antioxidant enzyme activities while freezing sensitivity was increased in transgenic tomatoes (**Figure 6**). Our results suggest that the *PsHAT5* transcription factor has potential applications in drought and salt tolerance breeding.

## Data availability statement

The datasets presented in this study can be found in online repositories. The names of the repository/repositories and accession number(s) can be found in the article/**Supplementary Material**.

## Author contributions

XL and AL were co-first authors of the present paper and co-wrote paper. CL and YW completed the preparation of the trial materials and acquisition of data, SW completed the analysis of the trial data. JL and JZ are the co-corresponding authors of this paper and directed the experimental design. All authors reviewed the manuscript. All authors contributed to the article and approved the submitted version.

## Funding

National Natural Science Foundation of China (32160061) [Entry name: Study on improving water use efficiency of Saussurea involucrata (SIPIP2;7) through chloride ion signal pathway].

## Acknowledgments

We are grateful for the support of the research group during this research work.

## Conflict of interest

The authors declare that the research was conducted in the absence of any commercial or financial relationships that could be construed as a potential conflict of interest.

## Publisher’s note

All claims expressed in this article are solely those of the authors and do not necessarily represent those of their affiliated organizations, or those of the publisher, the editors and the reviewers. Any product that may be evaluated in this article, or claim that may be made by its manufacturer, is not guaranteed or endorsed by the publisher.
