# Supplementary material for: Overexpression of Pyrus sinkiangensis HAT5 enhances drought and salt tolerance, and low-temperature sensitivity in transgenic tomato
Source: Front Plant Sci. 2022 Nov 7;13:1036254. doi: 10.3389/fpls.2022.1036254 (PMC9676457; doi:10.3389/fpls.2022.1036254)
Supplement: Supplementary file 1 [file Table_1.docx]

>PsiHAT5

MSGGRVYGGGSSSGLSALFQNQRGSERLDSLFLSESSNSSSASFLGSRSMVSFEDVRGGSGSNRSLFRQYEHEDNGDDDLDEYLHQPGKKRRLTADQVQFLEKSFDVENKLEPERKVLLAKDLGLQPRQVAIWFQNRRARWKTKQLEKDYEELQANYNNLKADCESLSKENDKLKAEVTVLSDKLHLKEKEGGNSELSDTNRLSQEPPQKPIADTVSEGEVSKVSAVASKQEDLSSGRSDIFDSDSSHYTDAVHSSLLEPGDSSYAFEPEQSDLSQDEEDNFTKSLLPPYIFPKIEDVDYSDTPANSCNYAFPVEDHAFWSWSY

>PbrHAT5

MSGGRVYGGGSSSGLSALFQNQRGSERLDSLFLSESSNSSSASFLGSRSMVSFEDVRGGSGSNRSLFRQYEHEDNGDDDLDEYLHQPGKKRRLTADQVQFLEKSFDVENKLEPERKVLLAKDLGLQPRQVAIWFQNRRARWKTKQLEKDYEELQANYNNLKADCESLSKENDKLKAEVTVLSDKLHLKEKEGGNSELSDTNRLSQEPPQKPIADTVSEGEVSKVSAVASKQEDLSSGRSDIFDSDSSHYTDAVHSSLLEPGDSSYAFEPEQSDLSQDEEDNFTKSLLPPYIFPKIEDVDYSDTPANSCNYAFPVEDHAFWSWSY

>RcHAT5

MAGGGRVYSSSGGASFLLQNQRGSCAPEPLDSLFLSGSSNVSSTSPFLGSSMMSFEGVRRGNGLKRSAFQQYEHEDNCDEEFDEYFHQPGKKRRLTADQVQFLEKSFDLENKLEPERKILLAKDLGLQPRQVAIWFQNRRARWKTKQLEKDYDVLQADYNSLKADCDSICKENDKLKAEVVQLSDRLRLKDEEMANSEQSDTNKSNQEPQQMPIAESVSSEGEVSKLSVVACKEDLTSVKSDIFDSDSSHYTDGVHSSLLERGDSSYIFEPDQSDLSQDEEDNLSKTLLPPYIFPKLEDVDYTDPPANSCNFGFPVEDHAFWSWSY

>MdHAT5

MAGGRVYGGGSSSGLSALFQNQRGSERLDSLFLSGSSNSSSASFLGSRSMVSFEDVRGGSGSNRSLFRQFEHEDNGDDDLDEYLHQPGKKRRLTADQVQFLEKSFDVENKLEPERKVLLAKDLGLQPRQVAIWFQNRRARWKTKQLEKDYEELQANYNNLKANCESLSKENDKLKAEVTVLSDKLHLKEKERGNSELSDTNRLSQEPPQKPIADTVSEGEVSKVSAVASKQEDLSSGRSDIFDSDSSHYTDGVHSSLLEPGDSSYAFEPEQSDLSQDEEDNFTKSMLPPYIFPKIEDVDYSDTPANSCNYAFPVEDHAFWSWSY

>PpHAT5

MAGGRVYSGGASSSSLSVLLQNQRGPCASEPLDSLFLSGSSNSSSASPFLGSRSMVSFEDVRGGNGSNRSLFHQYEHEDNGDDDLDEYFHQPGKKRRLTVDQVQFLEKSFDMENKLEPERKILLAKDLGLQPRQVAIWFQNRRARWKNKQLEKDYEVLQANYNSLKADCESLAKENEKLKSEVLVLSEKLPHKEKESGTSELSDTNKVSQEPTQNPTADSLSECEVSKVSAVASKQEDLSSGKSDIFDSDSPHYTDGVHSSILEPGDSSYVFEPDQSDLSQDEEDNLSKSLLPPYIFPKLEDVDYSDTPVNSCNFGFPVEDHAFWSWSY

>PbHAT5

MSGGRVYGGGSSSGLSALFQNQRGSERLDSLFLSGSSNSSSASFLGSRSMVSFEDVRGGSGSNRSLFRQYEHEDNGDDDLDEYLHQPGKKRRLTADQVQFLEKSFDVENKLEPERKVLLAKDLGLQPRQVAIWFQNRRARWKTKQLEKDYEELQANYNNLKADCESLSKENDELKAEVTVLSDKLHLKEKEGGNSELSDTNRLSQEPPQKPIADTVSEGEVSKVSAVASKQEDLSSGRSDIFDSVSSHYTDAVHSSLLEPGDSSYAFEPEQSDLSQDEEDNFTKSLLPPYIFPKIEDVDYSDTPANSCNYAFPVEDHAFWSWSY

>PmHAT5

MAGGRVYSGGASSSSLSVLLQNQRGPCASEPLDSLFLSGSSNSSSASPFLGSRSMVSFEDVRGGNGSNRSLFHQYEHEDNGDDDLDEYFHQPGKKRRLTVDQVQFLEKSFDMENKLEPERKILLAKDLGLQPRQVAIWFQNRRARWKNKQLEKDYEVLQANYNSLKADCESLAKENEKLKAEVLVLSEKLPHKEKESGTSELSDTNKVSQEPTQNPTADSLSECEVSKVSAVASKQEDLSSGKSDIFDSDSPHYTDGVHSSILEPGDSSYVFEPDQSDLSQDEEDNLSKSLLPPYIFPKLEDVDYSDTPANSCNFGFPVEDQAFWSWSY

>PdHAT5

MAGGRVYSGGASSSSLSVLLQNQRGPCPSEPLDSLFLSGSSNSSSASPFLGSRSMVSFEDVRGGNGSNRSLFHQYEHEDNGDDDLDEYFHQPGKKRRLTVDQVQFLEKSFDMENKLEPERKILLAKDLGLQPRQVAIWFQNRRARWKNKQLEKDYEVLQANYNSLKADCESLAKENEKLKSEVLVLSEKLPHKEKESGTSELSDTNKVSQEPTQNPTADSLSECEVSKVSAVASKQEDLSSGKSDIFDSDSPHYTDGVHSSILEPGDSSYVFEPDQSDLSQDEEDNLSKSLLPPYIFPKLEDVDYSDTPVNSCNFGFPVEDHAFWSWSY

>Pu x PcHAT5

ALFQNQRGSERLDSLFLSGSSNSSSASFLGSRSMVSFEDVRGGSGSNRSLFRQYEHEDNGDDDLDEYLHQPGKKRRLTADQVQFLEKSFDVENKLEPERKVLIAKDLGLQPRQVAIWFQNRRARWKTKQLEKDYEELQANYNNLKADCESLSKENDKLKAEVTVLSDKLHLKEKEGGNSELSDTNRLSQEPPQKPIADTVSEGEVSKVSAVASKQEDLSSGRSDIFDSVSSHYTDAVHSSLLEPGDSSYVFEPEQSDLSQDEEDNFTKSLLPPYIFPKIEDVDYSDTPANSCNYAFPVEDHAFWSWSY

>PaHAT5

MVSFEDVRGGNGSNRSLFHQYEHEDNGDDDLDEYFHQPGKKRRLTVDQVQFLEKSFDMENKLEPERKILLAKDLGLQPRQVAIWFQNRRARWKNKQLEKDYEVLQANYNSLKADCESLAEENDKLKAEVLVLSGKLLHKEKESETSELSDTNKVSQEPPQNPTADSLSECEVSKVSAVASKQEDLSSGKSDIFDSDSPHYTDGVHSSILEPGDSSYVFEPDQSDLSQDEEDNLSKSLLPPYIFP

>GhHAT5

MAGGRVYRSNTSADAGSNNLSVLLQSQWVPSSSEPLDTLFIPGSSPSSFLGTRSMVSFEGVDRRRSYFRTFDEEEKVEEDIEEYLHRSEKKRRLTVDQVQFLEKSFEAENKLEPDRKVQLAKDLGLQSRQVAIWFQNRRARWKTKQLEKDYDSLQASYNSLKADYDNLVKETDKLKEEVVQLTDKLLLEGKEKGKSELPDAKTSSQELPSEAAEGEESKVVPVMKSDYTEGVHSSVLLEGAGSSYPFEPDQSDLSQDEEDNLSKGLLHLPSCVFPKLQDIDYSDPPAGSCNFGFPLDDHAFWSWAY
