# Supplementary material for: Overexpression of Pyrus sinkiangensis HAT5 enhances drought and salt tolerance, and low-temperature sensitivity in transgenic tomato
Source: Front Plant Sci. 2022 Nov 7;13:1036254. doi: 10.3389/fpls.2022.1036254 (PMC9676457; doi:10.3389/fpls.2022.1036254)
Supplement: Supplementary file 2 [file Table_2.docx]

Supplementary Material

**Supplementary Figures**


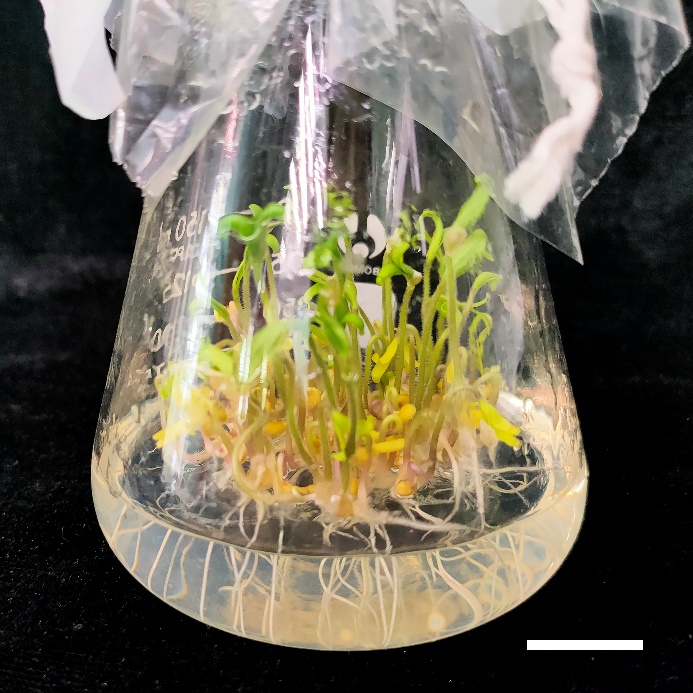


**Supplementary Figure S1.** The condition of 7 days after seed germination. The culture medium used was: 1/2 MS culture medium (2.2g MS powder, 15g sucrose, and 8g agar strip, make up to one liter with distilled water). pH5.8-6.0 Bar = 2cm.


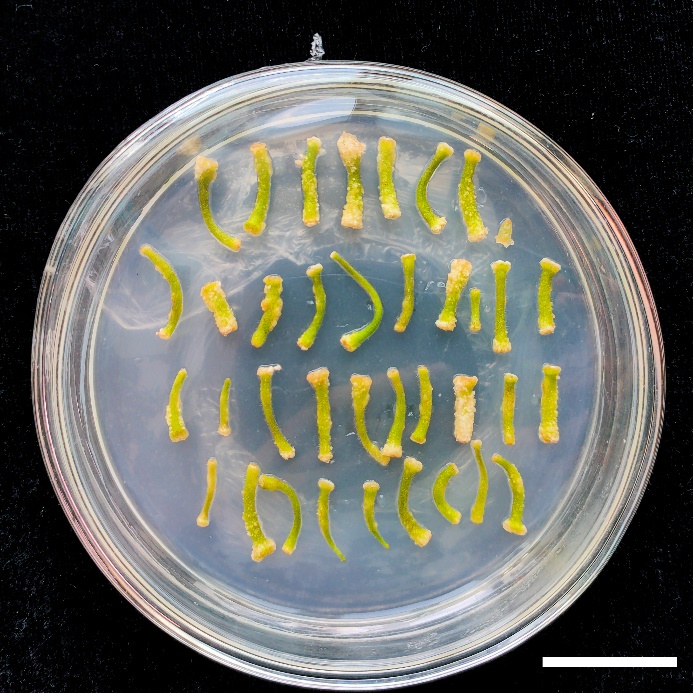


**Supplementary Figure S2.** Callus formation. Tomato hypocotyl was grown on MS medium supplemented with 2.0mg/L 6-BA, 0.3 mg/L IAA ,100 mg/L Kan, and 200 mg/L Tim. pH5.8-6.0 Bar = 3cm.


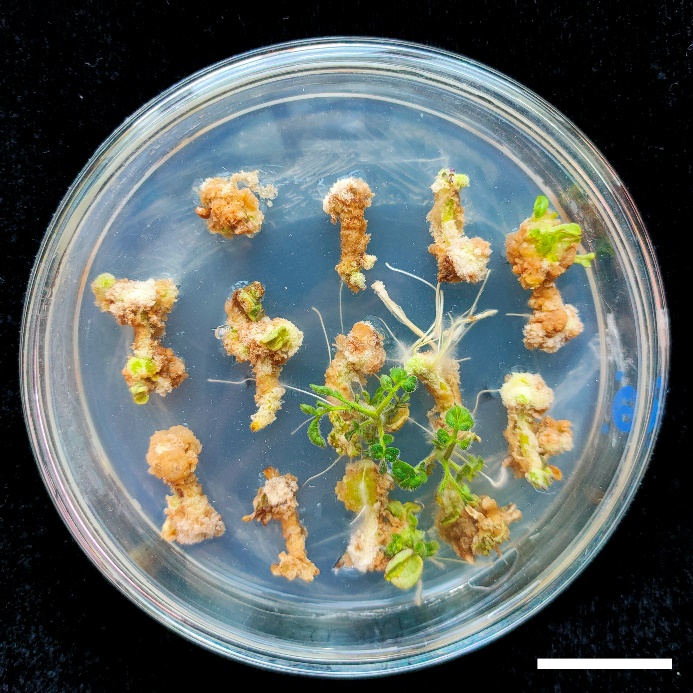


**Supplementary Figure S3.** Induction of indeterminate buds. The callus forms adventitious shoots on the induction medium for about 45 days. Bar = 3cm.

**
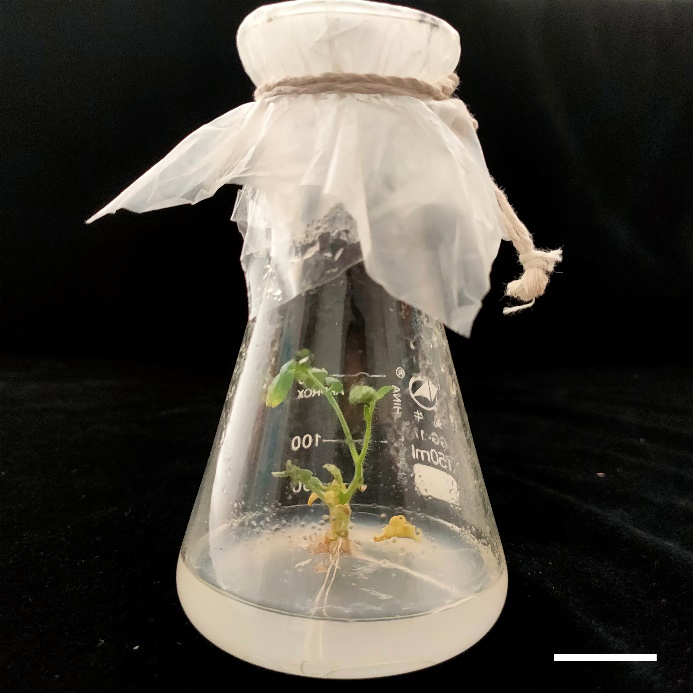
**

**Supplementary Figure S4.** Induced rooting. The formed adventitious shoots were grown on 1/2 MS culture medium containing 0.5 mg/L IAA, 100 mg/L Kan, and 200 mg/L Tim for rooting. pH5.8-6.0 Bar = 2.5cm.

**
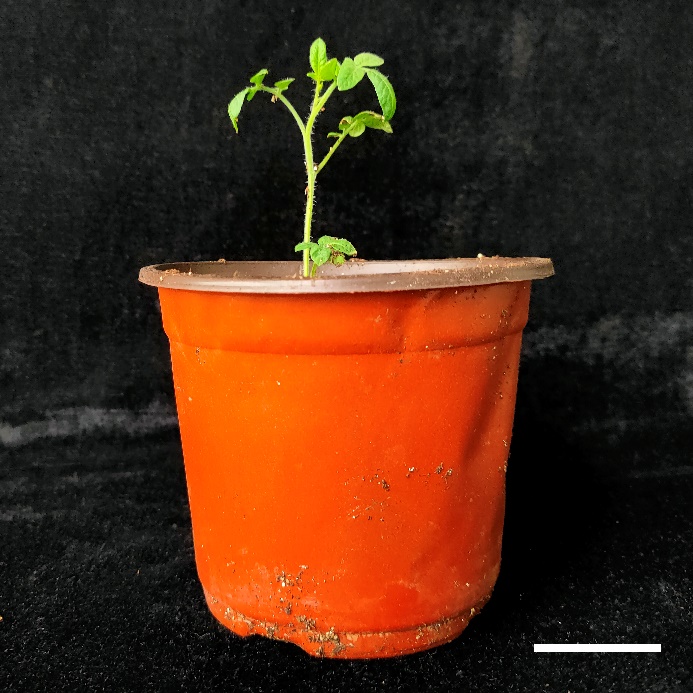
**

**Supplementary Figure S5.** Transplanting of mature seedlings. The volume ratio of cultivated soil, peat, vermiculite, and perlite we used was 3:1:2. (7.2cm inner diameter, 11.5cm high.) Bar = 3.5cm.
